# An interdisciplinary intervention for children and adolescents with multiple referrals and complex health complaints: a feasibility study

**DOI:** 10.1186/s12913-023-10250-y

**Published:** 2023-11-11

**Authors:** Ragnhild B. Lygre, Rolf Gjestad, Tone M. Norekvål, Stewart W. Mercer, Irene Bircow Elgen

**Affiliations:** 1 Department of Clinical Medicine, University of Bergen, Postbox 7804, 5020 Bergen, Norway; 2 Research Department, Division of Psychiatry, Haukeland University Hospital, Bergen, Norway; 3 Department of Child and Adolescent Mental Health Services, Haukeland University Hospital, Bergen, Norway; 4 Centre for Research and Education in Forensic Psychiatry, Haukeland University Hospital, Bergen, Norway; 5 Centre on Patient-Reported Outcomes, Haukeland University Hospital, Bergen, Norway; 6 Department of Clinical Science, University of Bergen, Bergen, Norway; 7 Usher Institute, University of Edinburgh, Edinburgh, Scotland

**Keywords:** Interdisciplinary, Multi-referral, Pediatrics, Mental healthcare, Patient reported experiences, Complex health complaints, Children, Adolescents, Parent

## Abstract

**Background:**

Children and adolescents with complex health complaints are often referred to several different healthcare specialists for assessments and treatment. This may result in fragmented care, higher risks of medical errors, and sub-optimal health outcomes. The aim of this non-controlled open label trial was to evaluate the feasibility of implementing a new interdisciplinary intervention for children and adolescents with multiple referrals and complex health complaints and to gather experiences from participating children, adolescents and parents.

**Methods:**

In all, 47 children and adolescents aged 6–16 years with multiple referrals at a tertiary hospital were invited to participate. The intervention was a half-day consultation based on a biopsychosocial model. The aim of the intervention was to clarify the child/adolescent’s condition(s) and provide a joint understanding and treatment plan in collaboration with the family. A team consisting of a pediatrician, a physiotherapist and a psychologist delivered the intervention. Acceptance and completion rate was recorded, and child- and parent-experience measures were collected; the children and adolescents completed the Visual Consultation and Relational Empathy Scale (CARE) five questions and parents completed two de novo created measures about their experiences.

**Results:**

Almost all invited families consented to participate (96%) and ultimately received the interdisciplinary intervention (92%). Mean age of the children and adolescents was 12 years, and under half were boys (40%). Before the intervention, 39 (91%) parents completed a questionnaire about previous experiences with healthcare. After the consultation 39 children and adolescents (91%) and 40 (93%) parents completed the questionnaire regarding their experience with the interdisciplinary intervention. Of the children and adolescents, 18–30 (47–77%) rated relational empathy in the intervention as “Very good” or “Excellent”. Of the parents, 35–39 (92–100%) rated their experience with the consultation using the more positive response options. The parents were significantly more content with the intervention compared to previously received healthcare (*p* < .001).

**Conclusions:**

The present intervention was highly acceptable with positively reported experiences from parents of, and children and adolescents with, complex health complaints. A future randomized controlled trial is required to test the effectiveness of this intervention.

**Trial registration:**

The study was registered at ClinicalTrials.gov NCT04652154 03.12.2020. Retrospectively registered.

## Background

Complex health complaints with compound mental and physical health challenges can appear as comorbidity, multimorbidity, medically unexplained symptoms or several diffuse health complaints that are difficult to disentangle, categorize, assess, diagnose and treat [1–5]. Such complaints are prevalent in both children and adolescents [2, 6–8], and several studies suggest an increasing trend [9, 10]. The expression and communication of health complaints in children and adolescents is affected by developmental level, resulting in a heterogeneous group of compound conditions [7]. Separating symptoms of general medical and mental conditions from medically unexplained conditions is a highly challenging, but important task [11].

Children and adolescents with complex health complaints are often referred to numerous medical specialists by their general practitioner for assessment and treatment [3]. Medically unexplained stomach pain accounts for more than 50% of consultations in paediatric gastroenterology [2]. The compound nature of these health complaints challenges the compartmentalized organization of specialist healthcare. The traditional way of organizing care—where specialists diagnose and treat symptoms and diagnoses independently – impede interdisciplinary assessments. Transitions between different departments and different specialists are vulnerable gaps in patients care trajectories, and require good information flow and collaboration between departments and health personnel, especially for children and adolescents with complex health complaints and multiple referrals to different hospital departments [1]. Failing to close these gaps, may result in fragmented care, increasing the risk of medical errors, and sub-optimal health outcomes [12, 13].

Fragmented care is a major shortcoming of modern healthcare delivery [14], and represents an unnecessary burden to the children/adolescents, their families and health services. Repetitive diagnostic testing, misdiagnosis, inadequate treatment, lack of unity among professionals, time spent away from normal everyday activities (child and parents) and different symptom explanations have an iatrogenic impact on these children’ and adolescent`s physical and mental health [2], and dramatic consequences for their societal participation [15]. These children and adolescents have significant impairments in school-, family, social- and physical activities [15], and these impairments are regarded as strong predictors of negative short- and long-term outcomes [16].

Thus, early and interdisciplinary interventions have the potential to alleviate the burden of health complaints, on both current and future quality of life and functional level. Several studies recommend tailored interdisciplinary interventions for children and adolescents with complex health complaints [17]. However, there is a lack of studies evaluating such interventions [12].

Researchers have developed an interdisciplinary half-day intervention, the Transitioning Patients Trajectories (TpT)- intervention, for children and adolescents with complex health complaints and multiple referrals transitioning between different departments in specialist heathcare [5]. This intervention was based on available research, user involvement, and pre-studies [1, 3–5] systematically analyzing care pathways, referral patterns, diagnoses and patient experiences with health services. The primary aim of the intervention was to clarify the child’ and adolescent’s compound condition, and to separate symptoms of general medical and/or mental conditions from medically unexplained conditions. The structure and theoretical background of the intervention was based on a biopsychosocial and systemic model for working with children with functional somatic symptoms and their families, developed by Kozlowska [18]. This model aims to understand the child’ and adolescent’s health complaints by connecting body, mind and social environment [19] and attempts to empower the child/adolescent and family by strengthening their own management of the condition.

It was unclear whether these children or adolescents and parents would agree to participate in the intervention, be able to complete the intervention, and report their experiences with it, right after. Thus, in line with the UK Medical Research Council guidelines for evaluating complex interventions [20], there was a need for a feasibility study in preparation for a future randomized controlled trial to test the effectiveness this intervention.

The primary aim of this study was to investigate the feasibility of an interdisciplinary intervention for children and adolescents with multiple referrals and complex health complaints in terms of acceptance and completion. The secondary aim was to evaluate child/adolescent and parent experiences of the intervention.

## Material and methods

### Design and setting

This feasibility study was designed as a non-controlled open-label trial of The Transitioning Patient Trajectories (TpT) intervention [5] for children and adolescents with complex health complaints and multiple referrals to specialist healthcare. The study took place from January 1^st^ 2020 to December 31^st^ 2022 at a tertiary referral hospital, Haukeland University Hospital, in Western Norway. Recruitment, intervention and all pre- and post-intervention measures were completed within this timeframe. This hospital provides healthcare to children and adolescents across a wide range of clinical specialties, including mental health. Its catchment area covers a population of about half a million inhabitants and serves a regional population of one million.

### Sample

Children and adolescents referred to the Section for Gastroenterology and Nutrition or the Section for Neurology and Habilitation at the Department of Pediatrics at Haukeland University Hospital were invited to participate in the study. Inclusion and exclusion criteria were based on prestudies [3–5].

Inclusion criteria:

- Age 6 to 16 years
- Three or more previous referrals
- At least one referral to the Department of Mental Health and one to the Department of Pediatrics at Haukeland University Hospital

Exclusion criteria:

- In need of emergency care
- Visits as part of inpatient treatment for specific diagnoses (such as epilepsy or Crohn’s disease)
- In special need for the regular scheduled consultation at the Department of Pediatrics

### Procedure

Every other week in the study period a study nurse extracted reports from the hospital record system for children and adolescents who were newly referred to the above-mentioned sections at the hospital. The Principal Investigator (the last author) assessed cases according to inclusion/exclusion. The study nurse contacted the parents/guardians of all eligible children and adolescents by telephone, inviting them to the study and offering them the TpT-intervention [5]. Families declining to participate received standard care (usually a scheduled appointment with a pediatrician) based on their referral at the department to which they were referred.

### The intervention

**Fig. 1:**
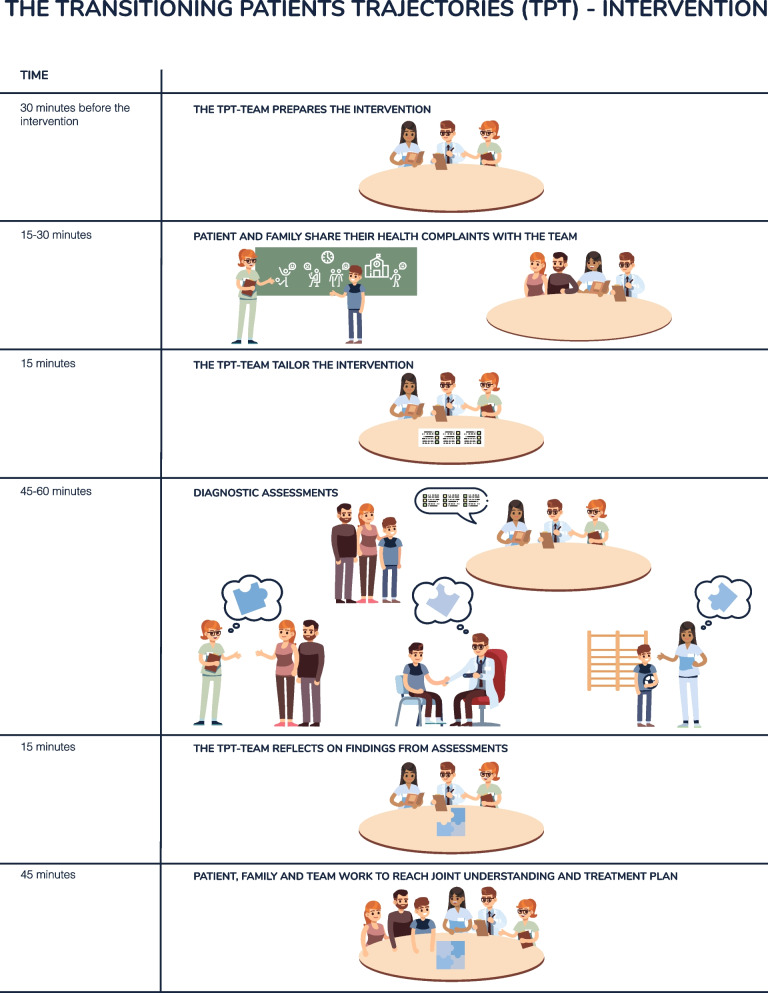
The structure of the TpT-intervention

Figure 1 presents the structure of the TpT- intervention.

On the day of the intervention, the family met the study nurse, reviewed the consent form, and provided written consent to participate in the intervention. The parents filled out questionnaires regarding the child’ or adolescent’s health status and their previous experiences with specialist healthcare, while the TpT-team prepared the consultation (Fig. 1). Then, the team introduced themselves and the structure of the intervention. The child/adolescent and parents shared their concerns and the child’ or adolescent’s health complaints with the team. This part of the intervention was an informal clinical interview and introduction to the child’s/adolescent’s biopsychosocial history, with emphasis on making the child/adolescent and parents feel comfortable, letting them talk freely, actively listening and forming a therapeutic alliance. A whiteboard was used as a narrative technique and conversation tool [21, 22]. Based on this clinical interview and the child’ or adolescent’s medical records, the team customized a program of assessments, intended to further clarify the child’s condition through completing the biopsychosocial model [21]. Typical assessments were paediatric physiotherapeutic examinations, such as Movement Assessment Battery for Children [23], somatic clinical examination and psychological interviews and assessments. Following these assessments, the TpT-team discussed the results of their assessments. Then the team had a joint summary of the day, and involved the family in reaching a joint biopsychosocial understanding of the child’/adolescent’s health complaints, and co-created a feasible and comprehensive treatment plan [21]. These plans included for instance psychoeducation and treatment related to a relevant disorder, referral to mental health or paediatric services for assessment of other disorders, or treatment focusing on coping and self-regulation. Both parents were encouraged to participate in the intervention, as studies suggest parental involvement could improve treatment outcomes for this group of children and adolescents [7]. Psychoeducation about the nature and management of health complaints was an overarching theme throughout the intervention. The families were also offered a follow-up consultation about 12 weeks after the TpT-intervention. The purpose of this follow-up consultations was first and foremost to check progress according to the agreed treatment plan, and if needed, repeat or review the joint understanding of the child or adolescent’s complaints or do additional assessments or measures.

### Therapists

Four teams delivered the intervention; two focusing on neurology and two on gastroenterology [3]. Each TpT-team consisted of a paediatrician, a physiotherapist and a psychologist [4, 5]. In total there were nine team members: four paediatricians, three physiotherapists and two psychologists. Each of these with considerable experience (> 10 years) in their field; paediatricians in gastroenterology and neurology, physiotherapists in assessing children and adolescents with both mental and somatic symptoms, and psychologists in health psychology [5]. The idea was to work as complementary teams, where the team members both use their joint and individual experience and competence in addressing, assessing and co-creating a joint and complementary understanding of the child’/adolescent’s health complaints. The teams were coached by an experienced team coach [5].

### Data collection

The number of families who were offered the intervention, and families that accepted, was recorded (acceptance rate). In addition, the number of children and adolescents completing the intervention (completion rate), and children, adolescents and parents reporting their experiences with the intervention, was recorded (measure completion rate). We used both child/adolescent- and parent-reported experience measures. The children/adolescents completed a patient-rated experience measure of the interpersonal quality of healthcare encounters immediately after the intervention. The parents completed two questionnaires: one directly before the intervention, and one immediately after the intervention.

### Measures

The feasibility of the intervention was measured using:

- Acceptance rate of the intervention
- Completion rate of the intervention
- Completion rate of the child/adolescent- and parent-reported experiences measures
- Child/adolescent- and parent-reported experience measures

#### Acceptance and completion rate

The current study assessed acceptability using objective measures such as retention/dropout, in line with the majority of studies assessing acceptability [24]. Acceptance rate for the intervention was measured recording the number of families offered the intervention that accepted. Completion rate of the intervention was measured recording how many of the families receiving the intervention that completed the full intervention. Completion rate of experience measures was measured recording how many of the children, adolescents and parents receiving the intervention, completed the child/adolecent- and parent-experience measures. This tested both acceptability and demand for the intervention [25].

#### Child/adolescent-reported experience measure

Patient perception of empathy is related to outcome of, and compliance to, treatment [26]. The Visual Consultation and Relational Empathy (CARE)-measure five questions (5Q) [27] is a patient-rated experience measure of the interpersonal quality of healthcare encounters. The measure is shown to have been valid and reliable in both primary and secondary care, among children, adolescents and adults and in several countries worldwide [28, 29]. The measure is found both feasible and acceptable to use in a routine paediatric setting [30], and of particular importance for patients with complex health complaints [31]. The children and adolescents were asked to rate the team in terms of their ability to make the child/adolescent feel happy and calm (item 1), asking questions and letting the child talk (item 2), listening and understanding (item 3), explaining things (item 4) and making a plan (item 5). Response options are based on a 5-point visual analog scale with scores from “not very good” to “excellent” and a “not applicable” option [27]. The measure is reported to have a high internal reliability (Cronbach’s alpha 0.88) when used with children and adolescents in specialist healthcare [26]. With permission from the developer of the CARE measure, we adapted the measure to provide an evaluation of the whole TpT-team, versus just one clinician, as originally designed.

#### Parent-reported experience measures

Some studies suggest that parent experiences are more strongly related to symptom reduction and functional improvement, than child and adolescent experiences [32]. Two questionnaires were developed for evaluating parent experiences; one concerning their experiences of previously received healthcare - patient reported measure before intervention (PREM0) and one concerning their experience of the TpT-consultation - patient reported measure after intervention (PREM01). Both PREM0 and PREM01 was de novo created, developed for the purpose of the present study. The measures were developed in collaboration with the Centre on Patient-Reported Outcomes at Haukeland University Hospital, parents of children receiving the TpT-intervention and adolescents from the Youth Council at the hospital. Before the intervention, parents were asked five questions regarding previous experience with specialist healthcare (PREM0), scored on a 4-point scale [1–4]. Scores ranging from 3–4 indicates a more positive experience, while scores 1–2 indicates a more negative or neutral experience. Right after the intervention parents received a questionnaire consisting of five questions regarding their experiences of the TpT-intervention (PREM01). The questions were also scored on a 4-point scale [1–4]. Scores ranging from 3–4 indicates a more positive experience, while scores 1–2 indicates a more negative or neutral experience.

### Progression criteria

The following criteria for evaluating the feasibility of progression to a randomised controlled trial (RCT) of the TpT-intervention was based on previous studies and the chosen feasibility measures [1, 3–5]:

- Acceptance rate of the intervention of > 80%.
- Completion rate of the intervention of > 80%.
- Completion rate of the child/adolescent- and parent-reported experiences measures: > 80% in line with previous studies on the use of CARE [26].
- Child/adolescent- and parent-reported experience measures: More than 50% of responses in the two higher response categories for the Visual CARE measure and the parent-reported experience measure of the intervention (PREM01).
- A significant difference in parent-reported experience between previous experience with healthcare and experience with the TpT-intervention, to suggest that this is a possible improvement of existing healthcare for children and adolescents with complex health complaints and multiple referrals.

In addition, the feasibility of the study was evaluated using Shanyinde et al. [33] suggestion of methodological issues that should be evaluated in the context of a future RCT, and Bugge et al.s [34] algorithm for decision-making after pilot and feasibility trials (ADePT) to suggest possible solutions to problems revealed by the feasibility study, and inform further decision making.

### Data analyses

Descriptive analyses were used to describe the child/adolescent- and parent-experience measures in terms of median, frequencies and valid percent. The response option “Does not apply” in the CARE measure was treated as missing in the analyses. Normality was explored with skewness and kurtosis, and homogeneity in variances with the Levene’s test. A paired sample T-test was used to test for differences in parent experiences with previous healthcare (before the intervention) and parent experiences with the interdisciplinary intervention (right after the intervention). Bootstrapping was used as a sensitivity analysis. This is a robust method regarding the distributional form of the variable. Statistical significance level was set to *p* < 0.05. The analyses were performed in SPSS version 29.0 [35]. Data are interpreted only as feasibility data.

### Ethical considerations

The study was approved by the Norwegian Regional Committee for Medical and Health Research Ethics 16.04.2018 (REC 2018/344) and retrospectively registered 03.12.2020 on www.clinicaltrials.gov (ID NCT04652154). The parents consented orally via telephone, and written consent was further obtained from the parents directly before the intervention. Informed consent was obtained from all participants legal guardian(s). The children were informed about their participation, but written consent from the children was not obtained, in reference to the approval from the Norwegian Regional Committee for Medical and Health Research Ethics (REC 2018/344). Children aged 6–12 years were age-appropriately informed about their participation and adolescents aged 12–16 years received written information about the project.

## Results

**Fig. 2:**
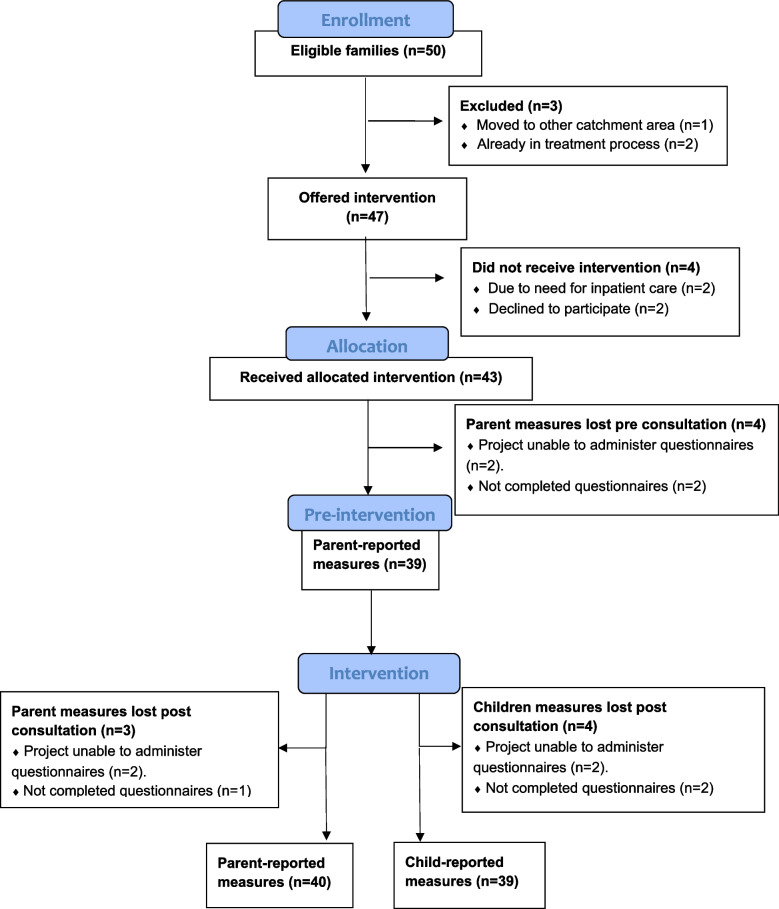
Flow chart of included children/adolescents and parents

Mean age of the included children and adolescents was 12 years and three months, and 40% of the children and adolescents were boys. Of the children and adolescents, 50 were eligible and 47 (96%) families were invited to participate, one family moved, and two families were already in an outpatient treatment process (Fig. 2).

### Acceptance and completion rates

Of the 47 invited families, 45 consented to participate (96%), and 43 out of these 45 (96%) received the intervention (Fig. 2). Of these 43, 39 (91%) parents completed the questionnaire before the intervention, 39 children and adolescents (91%) and 40 (93%) parents completed the questionnaires after receiving the intervention. Due to logistic reasons, only 41 families were presented with the questionnaires. In total, 43 out of 50 (86%) eligible families completed the intervention, and 39 (78%) children and adolescents and 40 (80%) parents out of the 50 eligible families completed the evaluation measures. 27 out of 43 (62,8%) patients were originally referred to the Section for Neurology and Habilitation and met the neurology TpT-team, and 16 out of 43 (37,2%) were originally referred to the Section for Gastroenterology and Nutrition and met the gastroenterology TpT-team.

### Child/adolescent- and parent-reported experience measures

#### Child/adolescent-reported experiences of the interdisciplinary intervention

Of the 47 families offered the TpT-intervention, 39 (83%) children and adolescents reported their experiences of the intervention using the Visual CARE measure 5Q. Of the children and adolescents 38 responded to all items, with only two (2%) “Does not apply” responses for two items. There was one missing response to item 5. The five questions for the children and adolescents are presented in Table 2 with median and valid percent. The single item response median scores from the Visual CARE measure 5Q were 4 (Very good) for items 1–4, and 3 (Good) for item 5 (Table 1). On each of the items 18–30 (47%-77%) of the responses were in the higher categories (4 = Very good, 5 = Excellent). Skewness on each item was found to have values between -0.1 and 1.5. Kurtosis on each item was found to have values between -0.9 and 7.1. Mean CARE sum score was 19 points out of a maximum possible score of 25 (SD: 3.3 points), with skewness and kurtosis at -0.2 and -0.8 respectively.

**Table 1:** Distribution of the Visual CARE measure 5Q scores on the TpT-intervention

Items	N	Median	n (%*)	n (%*)	n (%*)	n (%*)	n (%*)	n (%*)
			Not very good	Ok	Good	Very good	Excellent	Does not apply
How were the people you met today at…								
1. Making you feel happy and relaxed?	39	4	-	1 (3)	13 (33)	17 (44)	8 (21)	-
2. Asking questions and letting you talk?	39	4	1 (3)	1 (3)	8 (21)	18 (46)	10 (26)	1 (3)
3. Listening and understanding?	39	4	-	2 (5)	6 (15)	17 (44)	13 (33)	1 (3)
4. Explaining things?	39	4	-	3 (8)	13 (33)	13 (33)	10 (26)	-
5. Making a plan?	38	3	1 (3)	7 (18)	12 (32)	10 (26)	8 (21)	-

^*^ Valid percent

#### Parent-reported experiences of previously received healthcare

Out of 43 parents, 39 (91%) reported their experiences of previously received healthcare. The single item response median scores were 2 for all items. The valid percentages showed most responses, 15–23 (40–59%), in the second lowest category, indicating somewhat negative experiences with previously received healthcare. One respondent had a missing response on item 1 (Table 2). Skewness was between -0.7 and 0.8 on all items. Kurtosis was between -2.1 and -0.3 on all items.

**Table 2:** Distribution of scores on measure for parent reported experiences of previously received healthcare (PREM0)

Items	N	Median	n (%*)	n (%*)	n (%*)	n (%*)
			Poor	Fair	Good	Excellent
1. What is your experience of the healthcare your child has received until today?	38	2	9 (24)	15 (40)	13 (34)	1 (3)
			**None**	**A few**	**Most**	**Almost all**
2. To what degree has this healthcare satisfied the needs of your child?	39	2	4 (10)	20 (51)	13 (33)	2 (5)
			**Unsatisfactory**	**Partly satisfactory**	**Mainly satisfactory**	**Very satisfactory**
3. How satisfied are you with the extent of the healthcare your child has received?	39	2	2 (5)	19 (49)	17 (44)	1 (3)
			**No, made it worse**	**No**	**Yes, some**	**Yes, to a large degree**
4. Do you think previous healthcare has helped your child to deal with its complaints/problems in a better way?	39	2	-	23 (59)	15 (39)	1 (3)
5. Do you think previous healthcare has helped you to deal with your child’s complaints/problems in a better way?	39	2	-	20 (51)	19 (49)	-

^*^ Valid percent

#### Parent-reported experiences of the interdisciplinary intervention

Out of 43 parents 40 (93%) reported their experiences with the interdisciplinary intervention. The single item response median scores ranged from 3 to 4. Most of the parent responses to the single items, 35–39 (92–100%), were in the higher categories 3 and 4, indicating positive experiences with the interdisciplinary intervention. Missing responses on single items ranged from 1 to 3 (Table 3). Skewness ranged between -1.5 and 0 and kurtosis between -0.6 and 0, except on item 3, where these values were found to be -3.3 and 9, respectively.

**Table 3:** Distribution of scores on measure for parent-reported experiences of the TpT-intervention (PREM01)

Items	N	Median	n (%*)	n (%*)	n (%*)	n (%*)
			Poor	Fair	Good	Excellent
1. What is your experience of the healthcare your child has received today?	39	4	-	3 (8)	16 (41)	20 (51)
			None	A few	Most	Almost all
2. To what degree has this healthcare satisfied your expectations?	38	4	-	3 (8)	13 (34)	22 (58)
			Unsatisfied	Mainly unsatisfied	Partly satisfied	Very satisfactory
3. How satisfied are you with meeting a team consisting of a physiotherapist, a psychologist and a doctor?	38	4	-	-	3 (8)	35 (92)
			No confidence	Some confidence	Large confidence	Very large confidence
4. How confident are you that this team can help finding a treatment to help your child with its health complaints?	40	3	-	3 (8)	18 (45)	19 (48)
			No, for sure	No, don’t think so	Yes, think so	Yes, for sure
5. If a friend needed similar help, would you recommend this intervention for him or her?	38	4	-	-	8 (21)	31 (80)

^*^ Valid percent

#### Parent-reported experience of previously received healthcare versus experience of the interdisciplinary intervention

Comparing parent-reported experiences of healthcare received before the interdisciplinary intervention with their experience of the interdisciplinary intervention indicated a significant difference in terms of parents being significantly more content with TPT compared to previous healthcare (Table 4).

**Table 4:** Bootstrapped differences in scores on PREM0 and PREM01

	Mean	Std. Deviation	Std. Error Mean	95% Confidence Interval (lower/upper)	Sig
PREM01 Sum—PREM0 Sum	1.26	.49	.08	1.10/1.42	< .001

### Feasibility analysis

The feasibility was also assessed according to previously proposed methodological issues in need for evaluation in the context of an RCT [33]. This analysis revealed three methodological issues that needs to be addressed: small sample, the lack of a control group, and thereby, the lack of randomization.

## Discussion

This is the first feasibility study of an interdisciplinary intervention for children and adolescents with multiple referrals due to complex health complaints. According to the predefined criteria for feasibility, the intervention is feasible and acceptable to the families in question. The number of families that accepted the intervention (acceptance rate), and the number of families accepting the intervention and completing it (completion rate), was more than 90%. All families receiving the intervention were able to complete the intervention, and the majority of both children and adolescents and parents were able to report their experience.

The number of missing or response option “Does not apply” were low. This suggest that it is possible to rate a whole team of three health professionals using the Visual CARE Measure 5Q, and not just one professional, as in previous studies [26, 30]. The results suggest that this intervention and its evaluation measures are acceptable and feasible for the target group. The evaluation measures also suggest that both parents, children and adolescents experience this intervention as very good, and that the parents view it as an improvement of existing healthcare.

### Acceptance and completion

The term acceptability is defined in different ways when it comes to healthcare interventions [24]. Definitions range from operational definitions of acceptance, such as drop-out rates, discontinuation and patient satisfaction to more conceptual definitions of acceptability focused on how patients react to the treatment [24]. Sekhon et al. [24] reviewed the different definitions of acceptability and defined acceptability as a “ (…) multi-faceted construct that reflects the extent to which people delivering or receiving a healthcare intervention consider it to be appropriate, based on anticipated or experienced cognitive and emotional responses to the intervention ([24], p. 1)”. The majority of studies Sekhon et al. [24] reviewed, assessed acceptability using objective measures such as dropout, discontinuation and withdrawal rates, as the current study does [24]. The high acceptance rate of the intervention is in line with some previous studies of family-based interventions for children with functional somatic symptoms [36], but considerably higher than other comparable interventions [37]. The high acceptance rate in our study could be due to previous experiences with fragmented assessments and treatments, as previous studies suggest that many of these families call for clearer communication about the treatment plan, and more collaboration between different departments at the hospital [1, 4]. A longer consultation with a whole team could also be a more welcomed intervention for these families, as it might suggest a more thorough, interdisciplinary and holistic assessment of the child/adolescent’s condition. Early intervention and proper management for children and adolescents with complex and unexplained health complaints is thought to improve prognosis and prevent a lengthy and disabling course of illness. The TpT-intervention offers a coordinated, interdisciplinary and individually tailored intervention for complex and unexplained health complaints in children and adolescents, with prospects to increase quality of life and social participation. An interdisciplinary intervention can embrace the complexity of compound conditions and provide multi-referred children and adolescents with effective treatment and more seamless care pathways. The high completion rate of the intervention could be because the intervention was performed in one day only.

### Child/adolescent-and parent-experience measures

As most of the responses on the child/adolescent-experience measure (CARE) (47–77%) are “Very good” or “Excellent”. This suggest that the children and adolecents felt that the team was empathic; mostly very good at making them feel happy and relaxed, asking questions and letting the child talk, listening and understanding and explaining. However, only 47% of the children reported that they experienced the TpT-team’s ability to make a plan as “Very good” or “Excellent”, which is below the progression criteria of 50%. This might suggest that the team should take greater care to ensure that the child or adolescent is age-appropriately included in the creation of the treatment plan and take measures to facilitate the child or adolescent’s acceptance and understanding.

The rate of not completed CARE measures (5%) and “not applicable” response options (3%) is in line with a study using the CARE measure in pediatric emergency care [26]. The rate of acceptance of the CARE measure is also comparable to this study, but lower than a preliminary evaluation of the Visual CARE measure 5Q in Scotland [30]. This preliminary study had both a broader setting and population (different somatic departments in three NHS health boards) and a broader age group (7–18 years). The study of CARE in pediatric emergency care [26] also shows a more positive rating of empathy in health personnel from children aged 7–11 years, than the current study. Here, however, the CARE measure was used to rate a team of three health professionals, compared to one physician in the study evaluation [26]. This could be due to differences in patient population, or the fact that we ask the child and adolescent to evaluate a team of three professionals, compared to just one professional. One could hypothesize that children and adolescents with complex health complaints may be more discouraged based on their previous experiences with healthcare. However, the fact that we have not asked the children and adolescents about their previous experiences of healthcare, nor included a control group, makes this hypothesis hard to verify. There might also be a difference between an emergency care setting and the current outpatient setting, in which patients in need of emergency care were excluded. Future studies should include children and adolescent’s previous experiences with healthcare and a control group to strengthen their results. The parent-reported experiences with the child’s previous healthcare indicate that the parents are less satisfied with previously received healthcare. However, it is unclear if this also applies to the children and adolescents.

The parents had a high completion rate of experience measures, pre- (PREM0) and post-intervention (PREM01). The parents reported relatively positive experiences of the interdisciplinary intervention, especially in terms of their evaluation of meeting a professional team and recommending the treatment to others. This is in line with studies in both pediatrics and mental health [38, 39]. However, some characterize parent reports of satisfaction as, at best, an overestimation of actual satisfaction [40]. This allows us to study, regardless of experience level, if this intervention constitutes a possible improvement of existing healthcare. Parents rated their experiences of the current intervention significantly more positive compared to previously received healthcare. However, retrospectively rating your experience of previous healthcare might be different from rating one intervention directly after receiving the intervention. Immediate enthusiastic responses might fade with time, if parents struggle to see any significant lasting results of the intervention. Several studies have also reported a relationship between recall period and respondent bias or error, however, the results are not consistent [41]. The lack of a control group limits the ability to control for such effects. The presence of a control group could also even out possible social desirability biases [42]. Adding qualitative data from interviews with both children and parents could also provide useful insight into mechanisms of change, and possibly further inform the selection of outcome measures in a future RCT.

### Strengths and limitations

The small sample size and no control group limits the generalizability of the results, however, they serve the purpose of the feasibility study, to inform a future RCT. The fact that just 40% of the sample were boys, might be a reflection of the composition of the sample, but it might also represent a skewness in the sample, in terms of gender. The use of self-developed questionnaires for parent-reported experiences with healthcare limits the ability to compare our results to similar studies. The use of different questionnaires for children and parents also limits the ability to compare their experiences. There was a small number of children and/or adolescents declining to fill out the experience measures. These children and/or adolescents might be dissatisfied, and thus these missing responses could possibly affect the reported experiences with the intervention negatively.

The strengths of the study are a large percentage of eligible families that consented to receiving and completing the intervention (91%). Due to logistic reasons, the proportion of missing data was 5% percent. This missingness was an artefact of the design and believed to represent missing completely at random [43]. This will therefore not affect the results. However, this is an important area of improvement in preparing for a full-scale trial of the intervention. Dropout pre-intervention was low (4%) and none of the families left during the intervention.

### Methodological issues that need evaluation in the context of a future RCT

Using the ADePT process [34] for identifying and addressing problems, and informing further decision making in pilot and feasibility trials, three solutions were proposed to solve the three problems arising during the present feasibility study. The problems regarding the lack of control group and randomization, is proposed solved by selecting an appropriate method for randomization and conducting a feasibility study using randomization of cases and controls. To adequately power a future RCT, the issue of small sample size is proposed solved by recruiting patients and controls from additional sections from the Department of Pediatrics, beyond the two sections selected for this feasibility study.

## Conclusions

In conclusion, this interdisciplinary intervention is both feasible and acceptable for children and adolescents with complex health complaints and multiple referrals to specialist healthcare, and a progression to a future randomized controlled trial is recommended to test the effectiveness and cost-effectiveness of this intervention.

## Data Availability

Restrictions apply to the availability of these data, which were used under license for the current study, and therefore are not publicly available. Data are however available from the authors upon reasonable request to corresponding author.

## Abbreviations

TpT: Transitioning Patient Trajectories
UK: United Kingdom
CARE: Consultation and Relational Empathy Scale
5Q: Five questions
PREM0: Parent reported experiences with previously received healthcare
PREM01: Parent reported experience with the interdisciplinary intervention
RCT: Randomised controlled trial
ADePT: Algorithm for Decision-Making after Pilot and Feasibility Trials
SPSS: Statistical Package for the Social Sciences
REC: Norwegian Regional Committee for Medical and Health Research Ethics
ID: Identification number
SD: Standard deviation
N: Sample size
n: Observations in sample
Std: Standard
Sig: Statistical significance
NHS: National Health Service
